# Supplementary material for: Geroprotective drug discovery with an AI-enabled assay for heat resistance
Source: bioRxiv. 2026 Aug 10:2026.08.10.743574. Preprint. [Version 1] doi: 10.64898/2026.08.10.743574 (PMC13640661; doi:10.64898/2026.08.10.743574)
Supplement: Supplementary file 1 [file NIHPP2026.08.10.743574v1-supplement-4.pdf]

bioRxiv preprint doi: https://doi.org/10.64898/2026.08.10.743574; this version posted August 10, 2026. The copyright holder for this preprint (which was not certified by peer review) is the author/funder, who has granted bioRxiv a license to display the preprint in perpetuity. It is made available under a CC-BY 4.0 International license.

Supplementary Figures

Fig. S1.

Comparison of SAP scores after a 9hr heat shock at 35°C from worms fed 4 or 2 μL of 50x concentrated OP50. (** p<0.01, * p<0.05, ns p>=0.05, t-test)

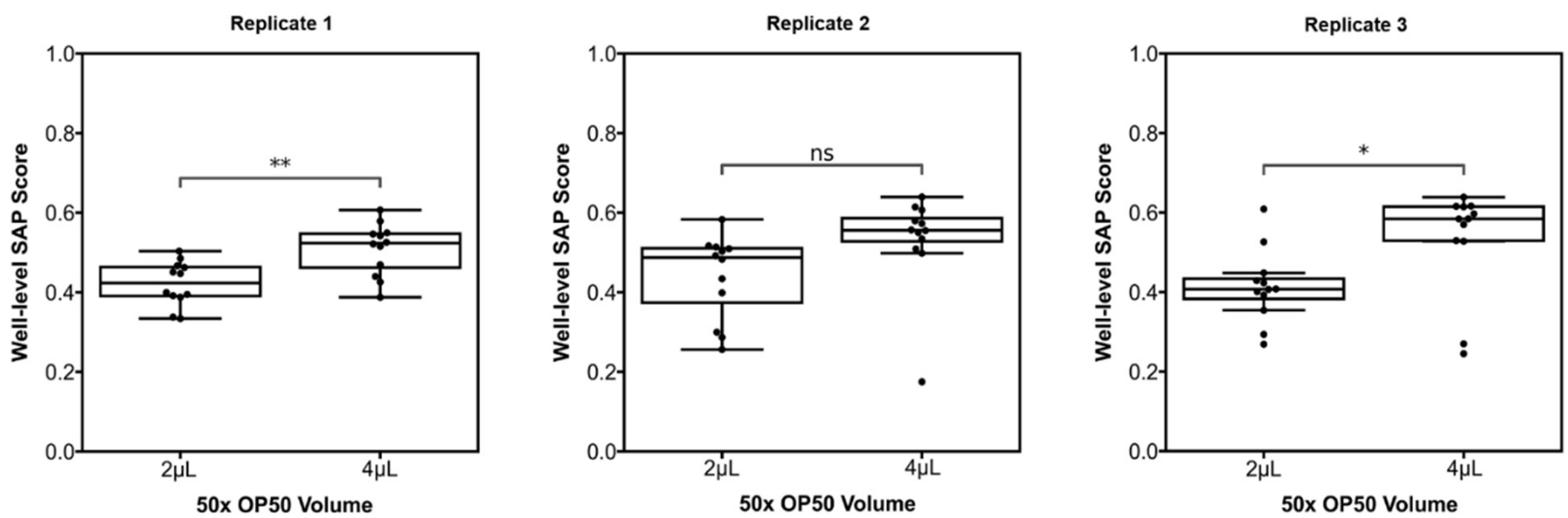

bioRxiv preprint doi: https://doi.org/10.64898/2026.08.10.743574; this version posted August 10, 2026. The copyright holder for this preprint (which was not certified by peer review) is the author/funder, who has granted bioRxiv a license to display the preprint in perpetuity. It is made available under a CC-BY 4.0 International license.

Fig. S2.

Timecourse experiments showing SAP scores of varied interventions compared to controls after heat shock from (a) age-1 worms, (b) 1mM tetracycline, (c) 1mM lipoic acid, and (d) 1mM trehalose. (**** $p<0.0001$, ****$p<0.001$, ** $p<0.01$, * $p<0.05$, ns $p>=0.05$, t-test).

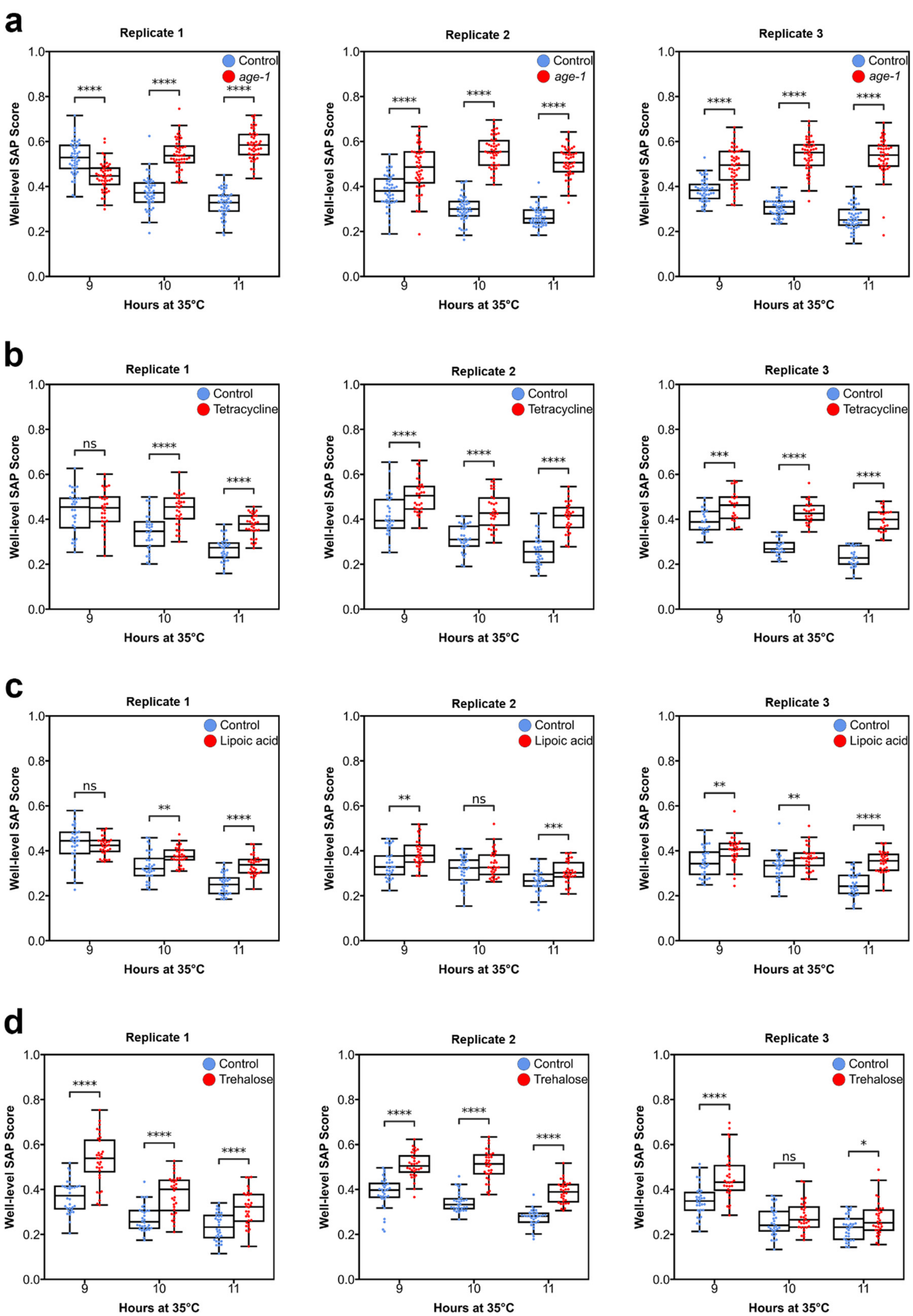

bioRxiv preprint doi: https://doi.org/10.64898/2026.08.10.743574; this version posted August 10, 2026. The copyright holder for this preprint (which was not certified by peer review) is the author/funder, who has granted bioRxiv a license to display the preprint in perpetuity. It is made available under a CC-BY 4.0 International license.

Fig. S3.

Dose response of hit drugs. 31 hit compounds retested at varying doses in the heat shock assay. SAP scores represent two-replicate averages.

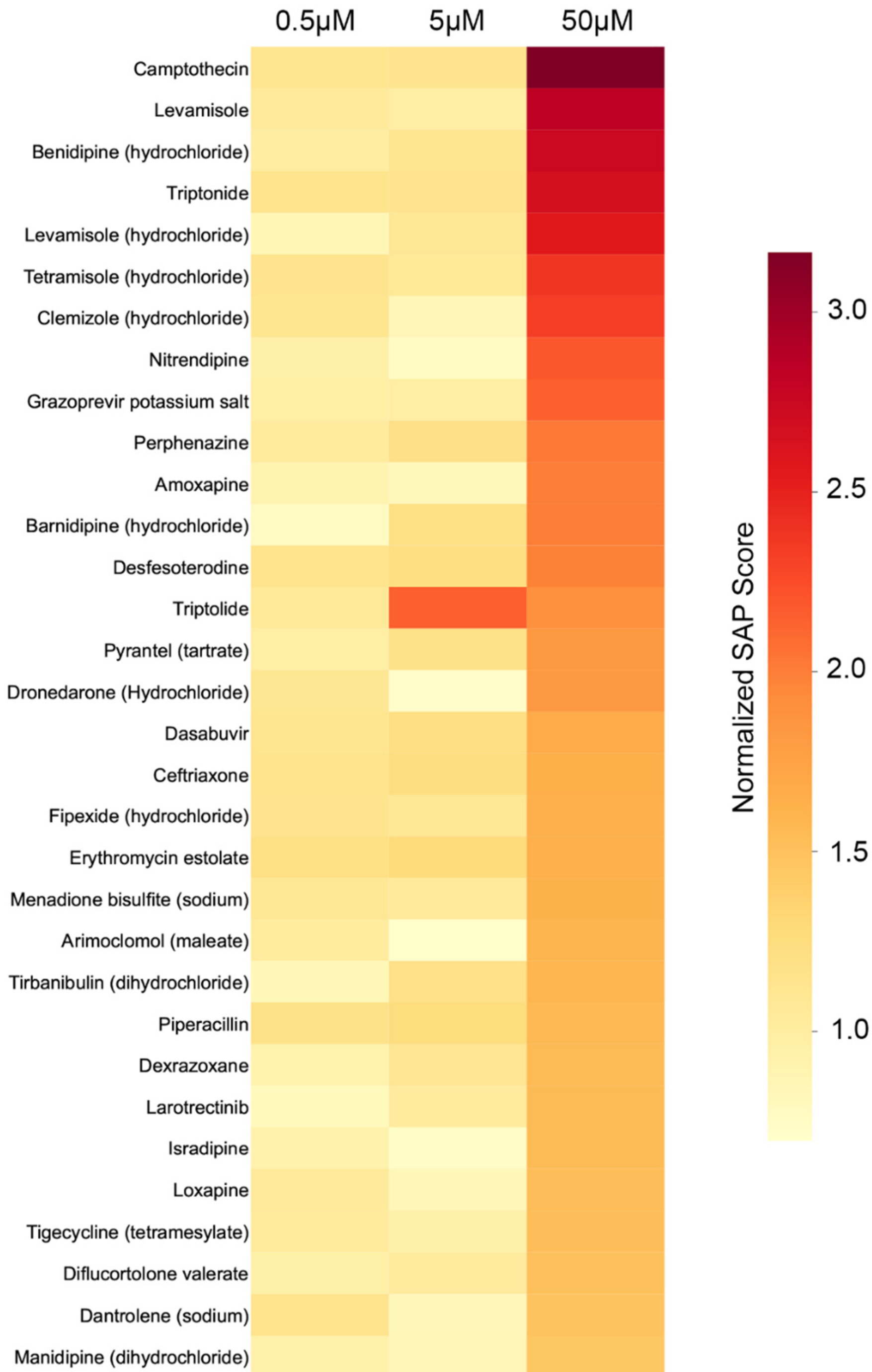

bioRxiv preprint doi: https://doi.org/10.64898/2026.08.10.743574; this version posted August 10, 2026. The copyright holder for this preprint (which was not certified by peer review) is the author/funder, who has granted bioRxiv a license to display the preprint in perpetuity. It is made available under a CC-BY 4.0 International license.

Fig. S4.

Longitudinal lifespan experiments. (a-f) Survival curves for all drugs. Each dotted line represents a separate biological replicate. Cox-proportional hazards test for significance (ns = not significant, * p<0.05, **** p<0.00005).

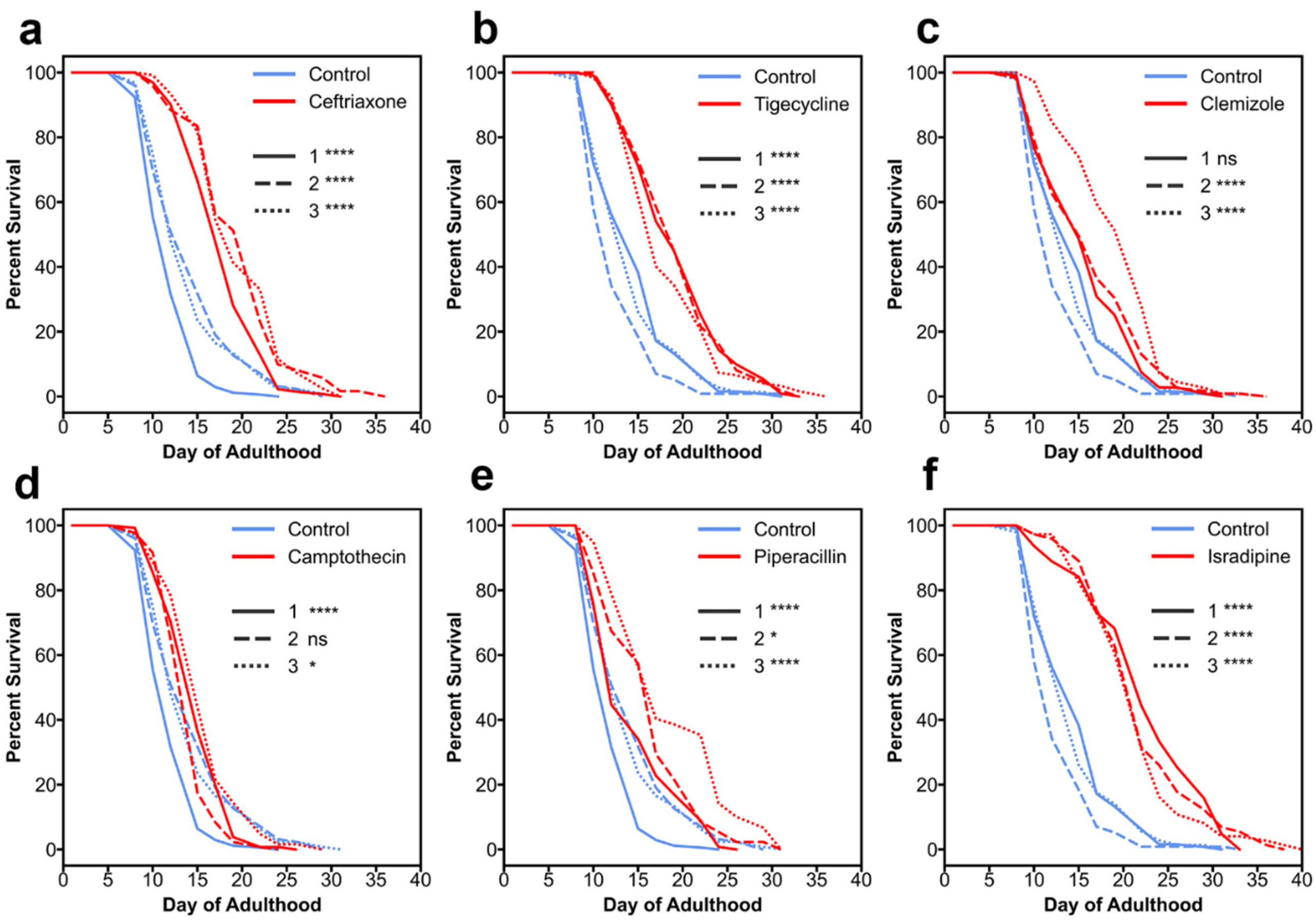

bioRxiv preprint doi: https://doi.org/10.64898/2026.08.10.743574; this version posted August 10, 2026. The copyright holder for this preprint (which was not certified by peer review) is the author/funder, who has granted bioRxiv a license to display the preprint in perpetuity. It is made available under a CC-BY 4.0 International license.

Fig. S5.

Life-extending drugs that are not protective in senescent HUVECs. (a-c) senescent cells versus proliferating cells. (d-f) senescent cells versus total cells. Error bars denote standard error of the mean.

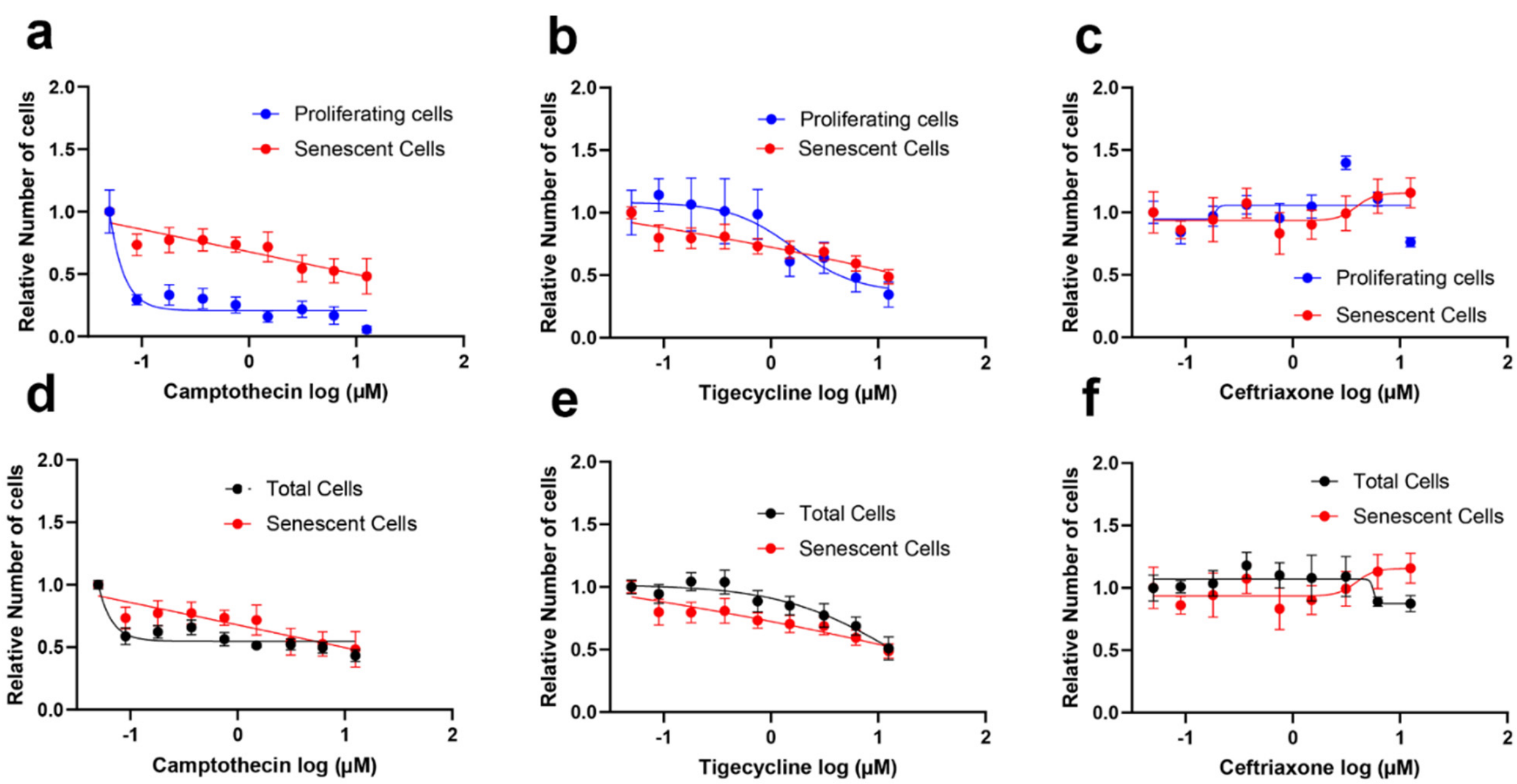

bioRxiv preprint doi: https://doi.org/10.64898/2026.08.10.743574; this version posted August 10, 2026. The copyright holder for this preprint (which was not certified by peer review) is the author/funder, who has granted bioRxiv a license to display the preprint in perpetuity. It is made available under a CC-BY 4.0 International license.

Table S1. Heat stress hits that extend longitudinal lifespan.

Column: 1) Drug name. 2) Target category provided by MedChemExpress. 3) Class provided by MedChemExpress. 4) Percent increase in mean lifespan of all recorded worms for an intervention. 5) Drug formulation molecular weight.

Name	Target	Class	% increase in mean lifespan
Ceftriaxone sodium	Antibiotic; Aurora Kinase; Bacterial; GSK-3	Anti-infection; Cell Cycle/DNA Damage; Epigenetics; PI3K/Akt/mTOR; Stem Cell/Wnt	43
Tigecycline tetramesylate	Antibiotic; Autophagy; Bacterial	Anti-infection; Autophagy	36.9
Clemizole hydrochloride	HCV; HCV Protease; Histamine Receptor; TRP Channel	Anti-infection; GPCR/G Protein; Immunology/Inflammation; Membrane Transporter/Ion Channel; Metabolic Enzyme/Protease; Neuronal Signaling	23
(S)-(+)-Camptothecin	ADC Payload; Antibiotic; Apoptosis; Fungal; Influenza Virus; MicroRNA; Topoisomerase	Antibody-drug Conjugate/ADC Related; Anti-infection; Apoptosis; Cell Cycle/DNA Damage; Epigenetics	12
Piperacillin sodium salt	Antibiotic; Bacterial; Beta-lactamase; Penicillin-binding protein (PBP)	Anti-infection	23.2
Isradipine	Autophagy; Calcium Channel	Autophagy; Membrane Transporter/Ion Channel; Neuronal Signaling	55.3

bioRxiv preprint doi: https://doi.org/10.64898/2026.08.10.743574; this version posted August 10, 2026. The copyright holder for this preprint (which was not certified by peer review) is the author/funder, who has granted bioRxiv a license to display the preprint in perpetuity. It is made available under a CC-BY 4.0 International license.

Extended Data

Data S1. Heat stress screen results for 2,782 FDA-approved drug library.

Data S2. Single-timepoint lifespan assay survival data for 31 final hit compounds.

Data S3. Longitudinal lifespan data for six top hits.
